# An unusual case of *aggregatibacter aphrophilus* liver abscess

**DOI:** 10.11604/pamj.2018.31.115.16409

**Published:** 2018-10-17

**Authors:** Ilias Papakonstantinou, Zoi Psaroudaki, Efstathia Perivolioti

**Affiliations:** 1 4^th^ Department of Internal Medicine, “Evangelismos” General Hospital of Athens, Athens, Greece; 2 Department of Clinical Microbiology, “Evangelismos” General Hospital of Athens, Athens, Greece

**Keywords:** Liver, abscess, immunocompetent, oropharyngeal, aggregatibacter aphrophilus

## Abstract

Liver abscess of oropharyngeal origin in an immunocompetent patient is a rare condition. Furthermore, microbiologic diagnosis of liver abscess can be challenging due to the tremendous diversity of the microorganisms implicated and culture difficulties under laboratory conditions. We report a case of a previously healthy 23-year-old male, who presented multiple liver abscesses, attributed to *aggregatibacter aphrophilus*, an obligatory oral gram-negative microorganism, that normally is a component of the commensal oral microbiota and non-virulent. The etiopathogenic microorganism was identified after needle aspiration of a liver abscess cavity. Treatment with broad-spectrum antimicrobials and percutaneous catheter drainage under computed tomography guidance of both abscesses, resulted in full recovery. *A. aphrophilus* represents a rare entity of liver abscess in healthy individuals and suggests that a pathogen of oropharyngeal origin should be suspected when an overt source of infection cannot be documented.

## Introduction

*Aggregatibacter aphrophilus* (*A. aphrophilus*), belonging to the *aggregatibacter* genus, is a gram-negative aerobic rod of the normal mixed oropharyngeal flora in humans, and most frequently represent a colonizer of oral mucocutaneous surfaces, like the supragingival plaque with no or limited pathogenicity [1,2]. Life-threatening invasive disease is rare. *Aggregatibacter spp* can be a prominent etiology of infective endocarditis and specifically *A. aphrophilus* exhibit an important cause of brain abscesses [1,3]. In this manuscript, we present a case of a previously healthy and immunocompetent person with *A. aphrophilus* liver abscess without predisposing conditions, which is an otherwise uncommon causative agent in healthy individuals.

## Patient and observation

**Figure 1:**
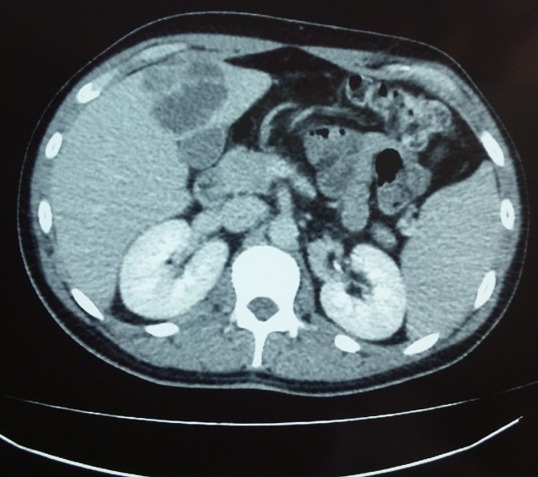
abdominal computed tomography (CT) showing an abscess in the left hepatic lobe

**Figure 2:**
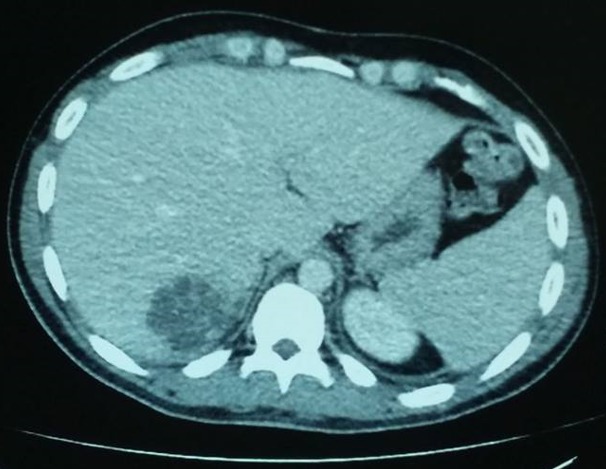
abdominal computed tomography (CT) showing an abscess in the right hepatic lobe

A 23-year-old previously healthy man, presented to the hospital's emergency department, with a 3-day history of high-grade fever up to 40°C and upper right abdominal pain. The patient's past medical history was non-significant. He was a student and had not received any medication. He had a pet dog and reported no recent travel. During physical examination on admission, vital signs were normal and he was febrile with a body temperature of 38°C. Abdominal examination revealed a tender palpable liver, with no signs of peritoneal irritation. The remainder of physical examination was unremarkable. Abnormal laboratory results on admission included a white blood cell count (WBC) of 20.830 (4-10.5x10³/μL), elevated C-reactive protein (CRP) level (28mg/dl, normal ≤0.5mg/dl), slightly elevated alanine aminotransferase (ALT) of 56 IU/L (normal 5-40 IU/L), and lactic dehydrogenase (LDH) of 255 IU/L (normal <225 IU/L). Alkaline phosphatase (ALP) was normal. Abdominal ultrasonography on admission revealed a mildly enlarged liver and spleen and the presence of 2 sizable, hypoechoic lesions. Abdominal computed tomography (CT) scan revealed the 2 aforementioned lesions in diameter of 5.6cm at the left liver lobe and 5.2cm in the right lobe Figure 1, Figure 2.

The lesions were hypodense and presented peripheral enhancement after intravenous contrast, similar to the morphology of hepatic abscesses. The patient was admitted for further evaluation and treatment. Culture guided antimicrobials and appropriate drainage was our first option. Empiric antibiotic therapy with intravenous ciprofloxacin (400mg every 12 hours), and metronidazole (500mg every 8 hours) was initiated. All cultures resulted negative. Initially, we performed percutaneous sonographic (U/S) guided aspiration into the left hepatic abscess cavity. A thick, purulent material of 40cc was drained and sent for culture. Microbiology revealed a Gram-negative bacterium that presented slow growth. On the 5^th^ and 8^th^ days of admission, we performed CT guided catheter drainage of both abscesses. Meanwhile irregular spikes of fever with daytime variation accompanied by night sweats persisted. Ciprofloxacin discontinued and meropenem at a dose of 1gr every 8 hours administered. Thorough screening for other possible sources of infection, resulted negative. Transthoracic echocardiography was negative for vegetations and showed mild mitral regurgitation. *Entamoeba histolytica* Abs, *echinococcus* Abs, *leismania* Abs, *brucella* Abs were negative. Gastrointestinal endoscopic evaluation was unrevealing. The patient was also HIV negative and no other immunodeficiency conditions were identified. Eventually, the isolate identified by the Vitek 2 automated system and classified as *A. aphrophilus*. To confirm the identification of the strain we performed matrix assisted laser desorption ionization-time of flight mass spectrometry (MALDI-TOF MS). The sensitivity test was performed with minimal inhibitory concentration (MIC) test strips; the isolate's susceptibility was to ceftriaxone ≤0.125 (S), cefepime ≤0.5 (S), ciprofloxacin ≤0.125 (S), gentamycin ≤0.5 (S), amikacin ≤0.125 (S) and to meropenem ≤0.032 (S). Percutaneous drainage of both abscesses, combined with antimicrobial treatment, was successful and the patient's clinical condition improved. Thankfully, no surgical procedure was required. The drains were removed immediately the fluid from the abscess cavities became clear. Meropenem was administered for a total of 2 weeks. Then meropenem was switched to oral ciprofloxacin for another 4 weeks and the patient was discharged from the hospital, in full recovery. Follow-up sonogram obtained almost 2 months after the drainage showed completely normal liver parenchyma without any residual liver cavities.

## Discussion

This is only one of the few reported cases of hepatic abscess formation by *A. aphrophilus* and one of the first in a healthy individual without significant oral pathology. An overview of 3 relevant cases with *aggregatibacter spp* related liver abscesses was found in the literature, and is presented on Table 1 [4-6]. *A. aphrophilus*, as the cause of liver abscesses in our patient, originated from the oropharyngeal region. The oral microbiome is abundant and composed with a diverse collection of organisms [7]. Among the major phyla that dominate bacterial oral community, the most important and predominant oral genera that are associated with liver abscesses include: *actinomyces*, *rothia* (*actinobacteria*), *prevotella*, *bacteroides*, *porphyromonas*, *capnocytophaga* (*bacteroidetes*), *fusobacterium* (*fusobacteria*), *veillonella* (firmicutes) and the *streptococcus millieri* group [7,8]. The metastatic mode of transmission to the liver, through hematogenous seeding of a distally located infectious source is relatively uncommon, especially if originating from the oral cavity [8]. *A. aphrophilus* initially localized the oropharynx and then under certain conditions, a covert bacteremia occured, leading to septic spread and metastatic abscess formation to the liver. Several mechanisms may contributed to evade this organism patient's host defense and cause an invasive infection e.g transient immunodeficiency from viral illness, possible alcohol consumption. A potentially zoonotic transmission of *A. aphrophilus* from his dog cannot be supported, since interactions of canine oral bacteria and the human's oral environment are difficult and the distribution of periodontopathic species in both humans and animals is generally different [9,10]. Distant infection and abscess formation is very likely to be associated with specific *A. aphrophilus* virulence properties, enabling immunological escape mechanisms. Ohyama et *al* have found through immunohistochemical staining, that periodontopathic bacteria can be carried in different organs [8]. From recent genome sequence research, Nørskov-Lauritsen remarks that *A. aphrophilus* express long filamentous fibrils and adhesins, that may participate in host colonization and potential opportunistic invasion of epithelial cells, proteolytic enzymes and toxins production are not mentioned though [1,2].

**Table 1:** reported cases of hepatic abscesses caused by *Aggregatibacter* species

References	Demographics Age (years), Gender	Predisposing Factors	Pathogen	Oral pathology	Localisation	Treatment	Outcome
Tsui 2012	30, Female	None, immunocom-petent	*Aggregatibacter aphrophilus* Identified with culturefrom pus	Recent minor dental procedure	Right liver lobe Single, hypodense lesion, 6.5 x 6 x 5cm	*Antimicrobials:* (5w in total) Initially ciprofloxacin + gentamicin, switched on 19^th^ day to ceftriaxone *Invasive:* Needle aspiration (x2, on2^nd^ and 9^th^ day)	Discharge after 5weeks Recovery
Ariyarat nam 2010	53, Male	None, previously healthy	*Aggregatibacter paraphrophilus* Identified from pus with polymerase chain reaction (PCR) amplification of the bacterial 16S ribosomal DNA and nucleotide sequencing	Recent dental root canal surgery	Right liver lobe Single mass, enhancing, with low attenuation, 4.5cm	*Antimicrobials* : (5w in total) Ertapenem + metronidazole shifted on 19^th^ day to oral amoxicillin *Invasive:* Percutaneous aspiration under ultrasound guidance on 6^th^ day followed by a percutaneous pigtail drainage	Discharge after 29 days Transferred to a tertiary hospital due brain abscsess formation
Sherlock 2005	52, Female	None, previously healthy	*Haemophilus (Aggregatibacter) aphrophilus* and *Mobiluncus Mulieris* Identified with culture from pus	Good, no recent dental work	Right liver lobe, posterior segment, sub- diaphragmatic Single mass, 5.5 x 6.5 cm	*Antimicrobials*: (5w in total) Amoxicillin/clavulanic + gentamicin, switched on 10^th^ day to orally ciprofloxacin + metronidazole *Invasive:* Percutaneous catheter drainage for 12days	Discharge after 3weeks Recovery

## Conclusion

In this report, we presented a case of multiple liver abscesses without any apparent contiguous or distal source of infection, attributed to an uncommon pathogen of oropharyngeal origin, the *A. aphrophilus*. The microbiologic diagnosis of *A. aphrophilus* was challenging due to slow growth under laboratory conditions. Treatment achived by combined use of antimicrobials with liver abscess percutaneous catheter drainage. This is one of the first reported cases of *A. aphrophilus* multiple liver abscesses in healthy persons, without underlying predisposing factors, suggesting that rare and under-recognized etiologic agents of liver abscess do occur. Physicians awareness and laboratory vigilance are required for appropriate detection and favorable outcome.

## Competing interests

The authors declare no conflict of interests.
